# Embryonic ethanol exposure and optogenetic activation of hypocretin neurons stimulate similar behaviors early in life associated with later alcohol consumption

**DOI:** 10.1038/s41598-024-52465-x

**Published:** 2024-02-06

**Authors:** Adam D. Collier, Nushrat Yasmin, Olga Karatayev, Abdul R. Abdulai, Boyi Yu, Milisia Fam, Samantha Campbell, Sarah F. Leibowitz

**Affiliations:** Laboratory of Behavioral Neurobiology, The Rockefeller University, 1230 York Avenue, New York, NY 10065 USA

**Keywords:** Motivation, Reward

## Abstract

The initiation of alcohol use early in life is one of the strongest predictors of developing a future alcohol use disorder. Clinical studies have identified specific behaviors during early childhood that predict an increased risk for excess alcohol consumption later in life. These behaviors, including increased hyperactivity, anxiety, novelty-seeking, exploratory behavior, impulsivity, and alcohol-seeking, are similarly stimulated in children and adolescent offspring of mothers who drink alcohol during pregnancy. Here we tested larval zebrafish in addition to young pre-weanling rats and found this repertoire of early behaviors along with the overconsumption of alcohol during adolescence to be increased by embryonic ethanol exposure. With hypocretin/orexin (Hcrt) neurons known to be stimulated by ethanol and involved in mediating these alcohol-related behaviors, we tested their function in larval zebrafish and found optogenetic activation of Hcrt neurons to stimulate these same early alcohol-related behaviors and later alcohol intake, suggesting that these neurons have an important role in producing these behaviors. Together, these results show zebrafish to be an especially useful animal model for investigating the diverse neuronal systems mediating behavioral changes at young ages that are produced by embryonic ethanol exposure and predict an increased risk for developing alcohol use disorder.

## Introduction

Early age of initiating alcohol use is one of the strongest predictors of a lifetime diagnosis of alcohol use disorder and a more severe, chronic course of use during adolescence^1,2^. Clinical studies have identified specific behaviors during early childhood that predict an increased risk for excess alcohol consumption during adolescence when the initiation and escalation of alcohol consumption often occur^3^. These early behaviors include hyperactivity and increased anxiety, novelty-seeking, risk taking/exploratory behavior, impulsivity, and alcohol-seeking, and they are associated with a later increase in alcohol consumption^4–7^. Notably, these same behaviors are also stimulated in adolescent offspring of mothers who consume alcohol during pregnancy^8–13^, and they are even detected early in children 9–10 years of age and, along with an increased sipping of 5% alcohol, are predictive of later alcohol use^14,15^.

Of particular interest is that these specific behaviors induced by early ethanol exposure and positively associated with later alcohol consumption are conserved across multiple species, from humans to rodents to zebrafish. Similar to clinical evidence, studies in pubertal or adult rodents prenatally exposed to ethanol have observed an increase in locomotor activity, anxiety, novelty-seeking, exploratory behavior, impulsivity, and alcohol-seeking, together with greater alcohol intake^16–20^. Also, in zebrafish, reports from this laboratory^21–23^ have shown that maternal consumption of ethanol increases locomotor activity and anxiety-like behaviors in adults as well as larval offspring, and other studies from this^22,24,25^ and other^26,27^ labs demonstrate that these behaviors are similarly stimulated by embryonic exposure to ethanol in the water at low-moderate concentrations, along with a later increase in voluntary consumption of alcohol.

There are a number of neurotransmitters and neuropeptides that mediate various behaviors associated with alcohol use disorder, including dopamine, γ-aminobutyric acid and melanin-concentrating hormone^28–30^. The hypocretin/orexin (Hcrt) peptide system in the hypothalamus, largely due to its widespread neuronal projections^31,32^ and neurochemical and genetic heterogeneity^33,34^, is well known to control numerous aspects of alcohol use disorder across multiple species^35,36^, including the different behaviors stimulated by embryonic ethanol exposure and associated with excess drug use^37,38^. Clinical studies have shown Hcrt levels in the cerebral spinal fluid to be positively related to anxiety^39^ and the blockade of Hcrt receptors to be effective in reducing anxiety symptoms associated with the use of and withdrawal from alcohol^40,41^ and in decreasing anxiety, panic and eating disorders^35^. In rodents, Hcrt increases the consumption of alcohol when centrally administered^42^, while blockade of Hcrt receptors or knockdown of Hcrt projections decreases alcohol drinking and alcohol-seeking behavior during dependence^36^. Also, prenatal ethanol exposure stimulates the proliferation, density and expression of Hcrt neurons^43,44^, and Hcrt is positively related to an increase in locomotor activity, arousal and anxiety^45,46^, with pharmacological and optogenetic studies also suggesting a role for Hcrt in impulsive behaviors^47^. Similarly, in zebrafish, embryonic exposure to ethanol increases the proliferation and density of Hcrt neurons, and these stimulatory effects on Hcrt neurons are accompanied by and positively correlated with an ethanol-induced increase in locomotor activity and anxiety-like behaviors along with an increase in voluntary consumption of ethanol-gelatin^24,37^.

With alcohol drinking in pregnant women on the rise over the past decade^48^, it becomes increasingly important to establish strong animal models that allow one to systematically investigate, using different techniques such as optogenetics, chemogenetics and laser ablation, the diverse neuronal mechanisms which are involved in controlling the repertoire of behaviors induced by embryonic ethanol exposure that predict susceptibility to develop alcohol use disorder. With the goal of developing such a model, we focused here on zebrafish, which exhibit sophisticated behaviors early in development that can be rapidly and efficiently studied using automated tracking software and are particularly useful for neurodevelopmental studies involving live imaging and other techniques difficult to perform in rodents^49^. Our first goal was to test at a young age, in larval zebrafish as well as pre-weanling rats for comparison, the effects of embryonic exposure to ethanol on the repertoire of early behaviors that are predictive of excess alcohol consumption, using a relatively low concentration of ethanol that does not produce physical dysmorphologies often reported at higher concentrations and thus provides a model of the highly prevalent alcohol-related neurodevelopmental disorder^50,51^. Building on evidence that embryonic ethanol exposure stimulates Hcrt neurons in the hypothalamus, our next goal was to use optogenetics in larval zebrafish to determine if the stimulation of Hcrt neurons has similar effects to those induced by embryonic ethanol exposure. In addition to suggesting the involvement of Hcrt in ethanol’s effects on these early behaviors, positive results would provide strong evidence supporting the use of zebrafish for investigating the different neuronal mechanisms mediating the behavioral changes induced by embryonic ethanol exposure that are predictive of later disturbances in behavior.

## Materials and methods

### Animals and housing

Adult female and male Sprague–Dawley rats, obtained from Charles River Breeding Laboratories, were maintained under standard lighting conditions (22 °C, 12:12-h light–dark cycle, with lights off at 8 am), with food (LabDiet Rodent Chow 5001) and filtered water available ad libitum. The breeding of rats was performed to strictly control the prenatal environment, and all timed pregnant rats were subsequently housed individually, weight matched, and randomly assigned to the experimental or control group. The sex of the offspring after birth was determined through visual inspection of the genital papilla and ano-genital distance as described^52^, allowing us to identify and test only the female offspring which are found to exhibit stronger behavioral changes after embryonic ethanol exposure than males^53^. In all experiments, 1 female rat offspring was used from each litter. The zebrafish examined in this report were the wildtype AB strain zebrafish and also the transgenic *Hcrt:ChR2-EYFP*^54^ zebrafish. Adult zebrafish were group-housed in 3 L tanks (Aquatic Habitat, Apopka, FL) with recirculating water flow at a temperature between 28 and 29 °C and a pH between 6.9 and 7.4, as previously described^55^. Adult zebrafish were fed once in the morning and once in the afternoon with live brine shrimp. Larval zebrafish were fed once in the morning with AP Zeigler diet according to their age. All the breeding and raising of animals for this study occurred within an AAALAC accredited facility using protocols approved by the Rockefeller University Institutional Animal Care and Use Committee and guidance of the NIH Guide for the Care and Use of Laboratory Animals. Also, this study was carried out in compliance with the ARRIVE guidelines.

### Embryonic ethanol treatment in rats and zebrafish

Pregnant rats were intraorally administered, from E10-E15 when Hcrt neurons develop in the hypothalamus^56^, with ethanol (30% v/v) at a low-moderate dose (2 g/kg/day) or a control solution of maltose-dextrin made isocaloric to the ethanol solution (control group), as previously described^44^. The daily dose of ethanol was split in half with all rats gavaged twice daily, with the first gavage occurring 2 h after start of the dark cycle and the second gavage occurring 7 h later. As reported in our previous publications, examination of the ethanol-exposed and control rats in this study revealed no differences in measures of the dams’ chow intake and body weight and of the size and body weight of their litters, with no spontaneous abortions. Also, with our prior studies of untreated groups repeatedly showing no differences from the control offspring, these untreated controls were not tested in the present study. Embryonic exposure of zebrafish to ethanol was performed, as described in our previous reports^10,27^. Briefly, zebrafish embryos, which were confirmed visually by multiple investigators using a developmental staging reference^57^ to be at the 26-somite stage at approximately 22 h post-fertilization (hpf), were removed from an incubator and placed in a solution of either 0.0% (control) or 0.5% (vol/vol %) ethanol for 2 h, a time period when the zebrafish hypothalamus and Hcrt neurons are just beginning to develop^55,58^. They were then washed in fresh embryo medium and returned to the incubator.

### Rat behavioral testing

A total of 4 separate groups of rats were tested for behavior, with behavioral testing conducted in a sound- and light-attenuated room (< 5 lx) starting 1 h into the dark cycle and different groups of animals used for each behavioral test. First, as previously described^59^, one group of rats was tested for 10 min at 15 days of age in a novel activity test chamber (43.2 cm × 43.2 cm) (Med Associates, Inc., St. Albans, VT, USA) to automatically measure locomotor activity as distance traveled (cm), thigmotaxis as an anxiety-like behavior and measured by distance traveled (cm) in the perimeter and number of entries to the perimeter, and exploratory behavior as number of rears and time (s) spent rearing. The perimeter was defined as a zone 5 cm from the edge of the activity chamber. In a second group of 15-day-old rats as previously described^60^, anxiety-like behavior was additionally evaluated within the light–dark preference test by inserting a dark box insert (21.6 cm × 43.2 cm) (Med Associates, Inc., St. Albans, VT, USA) into one half of the activity chamber and measuring time spent in the light zone of the environment, with reduced time in the light zone indicative of increased anxiety^60^. Then, in a third group of rats at 12 days of age when they are mobile and their eyes are still closed, we tested as previously described^61^ alcohol-seeking by measuring ethanol odor-induced locomotor activity in control and ethanol-exposed rats. Briefly, individual rats were first placed at one end of a black plexiglass runway (10 × 60 cm) covered in microfiber cloth that was placed atop a heating pad. A ruler was placed along the side of the runway, and the distance traveled (cm) during a 2 min period was manually scored. A cotton ball was moistened with either filtered water or filtered water containing 6% ethanol, and it was held immediately in front the rat’s snout for the full 2 min they were in the runway. If the rat moved down the runway, the investigator held the cotton ball in front of each rat’s snout without interfering with the rat’s behavior or making physical contact, and if the rat reached the end of the runway before the end of the 2 min period, the testing for that subject was completed. Finally, to measure their alcohol consumption during adolescence, a fourth group of female rats at 35 days of age were trained to voluntarily drink ethanol for 3 weeks using the 20% intermittent-access paradigm, as previously described^43,53^, and their 24 h intake of alcohol along with body weight was recorded three times weekly and averaged across the 3 week period, with intake expressed as g of ethanol consumed per kg of body weight.

### Zebrafish behavioral testing

All zebrafish behavioral testing occurred within a DanioVision chamber (Noldus, Wageningen, Netherlands), and activity was tracked by Noldus Ethovision XT 16 software, with environmental zones defined within the software. Different groups of zebrafish were used for each behavioral test, and all behaviors were tested starting 1 h into the light cycle before their regular feeding, to eliminate any stress possibly caused by the process of removing remnants of the food after feeding and any interference the food particles might cause with video tracking of the zebrafish. Control and ethanol-exposed AB larval zebrafish at 6 days post-fertilization (dpf) were individually transferred from their home tanks into a standard, 12-well culture plate containing fresh embryo media and immediately placed into the chamber where the different behaviors were tested. Control and ethanol-exposed zebrafish were evenly distributed within each behavioral testing plate. Locomotor activity was assessed in the first group by measuring distance traveled (cm) during a 20 min free swimming test. To assess anxiety-like behavior, thigmotaxis in this group was evaluated by measuring both the percent of time spent in the perimeter zone (defined as a zone 0.75 cm from the edge of each well) and the number of entries into the perimeter zone^36^, two measures together that enable us to rule out the possibility that alterations in thigmotaxis behavior are due solely to changes in locomotor activity or immobility. A third measure of anxiety, the absolute turn angle of each zebrafish, was also recorded to further understand the state of their anxiety^62^. Motor impulsivity was then measured as described^63^, by evaluating the number of swimming “peaks” defined as events when the fish traveled more than 0.5 cm in less than 12 s and by the distance traveled (cm) during each of these peaks. A second group of larval zebrafish was placed into individual wells of a new 12-well plate for a 20 min light–dark preference test, as measured by the percent of time spent in the light zone and entries into the light zone, with increased time and entries into the light indicative of increased anxiety-like behavior in larval zebrafish^37^. Exploratory behavior was next evaluated in a third group for 20 min as previously described^64^, by placing the larval zebrafish into a 12-well plate containing two 6-well environments that had channels between each of the 6 wells, allowing for free movement between each well, and was measured as the number of transitions between wells. Zebrafish were placed in the bottom middle well of each 6-well environment to measure exploratory behavior. Novelty-seeking behavior was examined in a fourth group for 20 min similar to that previously described^65,66^, by placing a novel object consisting of a 4 cm in diameter green colored piece of a plastic pipette tip into one side of the well of a 6-well plate with the object zone defined as a distance of 4 cm away from the object, and it was measured by the percent of time spent within the novel object zone and the number of entries into the novel object zone. Next, we evaluated alcohol-seeking behavior in a fifth group in a manner similar to novelty-seeking behavior, by placing in a 6 well plate a 75 μL sample of liquified plain gelatin mixed with 0.01 g of zebrafish AP 100 diet < 50 μM (Zeigler Bros, Gardners, PA, USA) (control-gelatin) at one end of each well and a 75 μL sample of liquified plain gelatin containing 1% ethanol as well as 0.01 g of zebrafish AP 100 diet < 50 μM (ethanol-gelatin) at the opposite end of the well, with the location of these samples alternated between the top and bottom sides of each well. Individual zebrafish were then added to each well and allowed to explore for 20 min, with alcohol-seeking measured as the percent time spent in and the number of entries into the ethanol-gelatin versus control-gelatin zones and preliminary tests showing control fish to spend a similar amount of time in each zone. Following behavioral testing, the fish were returned to their home tanks and given their daily feeding. Lastly, the consumption of alcohol in juvenile zebrafish was evaluated in a sixth group as previously described in adult zebrafish^21,22^, by manually quantifying the number of bites taken of a cube of either plain gelatin mixed with brine shrimp (control-gelatin) or plain gelatin mixed with brine shrimp and 1% ethanol (ethanol-gelatin). The zebrafish were first trained to consume control-gelatin from 24 to 29 dpf once per day and then fed either a cube of control- or ethanol-gelatin once at 30 dpf, and the number of bites were measured over a period of 15 min. Zebrafish were fed no food from 24 to 30 dpf other than the gelatin mixtures.

### Optogenetic activation of Hcrt neurons

To optogenetically activate Hcrt neurons in *Hcrt:ChR2-EYFP*^54^ zebrafish, we used an array of 6 blue and red LED lights (627 nm, MR-D0040-10S and 470 nm, MR-B0040-10S, respectively, Luxeon V-star, Brantford, Canada) mounted 18 cm above the well plates within the DanioVision chamber, with each color being applied at the same intensity and providing equal illumination across the well plate, as previously described^54^. Briefly, as has been described^54^, this *Hcrt:ChR2-EYFP* transgenic line was generated in which the *hcrt* promotor^67^ drives expression of channelrhodopsin-2 that is fused to EYFP, and thus ChR2 activates Hcrt neurons when exposed to the blue light but not the red light. Zebrafish were placed into their respective wells, and either red lights or blue lights were manually turned on for a period of 20 min during behavioral testing in larval zebrafish, for locomotor activity, anxiety-like behaviors, exploration, impulsivity, novelty-seeking, and alcohol-seeking, and for voluntary alcohol intake in juvenile zebrafish, as described above.

### RNAscope in situ hybridization and confocal microscopy

We used the RNAscope Multiplex Fluorescent Reagent Kit v2 (ACD Bio-Techne, catalog # 323100) to detect cfos transcripts in 6 dpf *Hcrt:ChR2-EYFP*^54^ zebrafish brains 20 min after either red light or blue light illumination using the Dr-hcrt (ACD Bio-Techne, catalog #887231-C1) and Dr-fosab-C2 probe (ACD Bio-Techne, catalog #446871-C2). The procedure included a combination of the manufacturer’s protocol and those reported in other studies^68,69^. Briefly, isolated brains from previously dehydrated fish were rehydrated in a 75%-50%-25% methanol-0.1% PBST series and fixed for at least 5 h in 4% PFA (in 1% Tween-20) at 4 °C. The samples were rinsed 3 times with 0.1% PBST and treated with Protease III for 15 min in a 40 °C water bath and washed again with 0.1% PBST followed by an overnight hybridization with the probe at 40 °C. The brains were then washed and post-fixed with 4% PFA for 10 min at room temperature. All subsequent incubations were done at 40 °C, with 3 × 15 min washes using 0.2 × SSCT between each step. They were then incubated with AMP1 (30 min), AMP2 (15 min) and AMP3 (30 min) in this order. To develop the Hcrt signal, samples were incubated in RNAscope Multiplex FL v2 HRP-C1 for 15 min, stained with Opal 570 (Akoya Biosciences FP1488001KT) in 1:1500 dilution in TSA buffer for 30 min, and blocked in the RNAscope Multiplex FL v2 HRP blocker for 15 min. The same steps were followed to develop cfos signal using the Opal 690 dye (Akoya Biosciences FP1497001KT) and RNAscope Multiplex FL v2 HRP-C2. Finally, the samples were incubated for 30 min at 4 °C in DAPI (1:200) diluted in 0.2% PBST, washed 3 × 5 min with PBS, and stored at 4 °C in fresh PBS until imaging. The RNAscope brain samples were imaged, beginning from the dorsal side of the brain, with a 40× objective lens on an inverted Zeiss LSM 780 laser scanning confocal microscope with a z step of 1.0 μm, and they were then visualized in Imaris 9.9.1 software. Five brains were imaged per condition.

### Statistical analyses

Data derived from the rat behavioral tests, for locomotor activity, thigmotaxis, light–dark preference, exploration, and alcohol-seeking in pre-weanling rats and for alcohol intake in adolescent rats, were analyzed by unpaired *t* tests with the Holm-Sidak multiple comparisons correction as necessary. Data derived from the zebrafish behavioral tests, for locomotor activity, thigmotaxis, light–dark preference, exploration, impulsivity, novelty-seeking and alcohol-seeking in larval zebrafish and for alcohol intake in juvenile zebrafish, were also analyzed by unpaired *t* tests with the Holm-Sidak multiple comparisons correction as necessary. Sample sizes for all rat behavioral tests ranged from n = 4–10, and sample sizes for the zebrafish locomotor and anxiety behavioral tests ranged from n = 50–80 and sample sizes for zebrafish exploration, impulsivity, novelty-seeking, alcohol-seeking and alcohol intake ranged from n = 10–40. Data were excluded from any zebrafish (~ 10% of the group) that remained entirely immobile throughout the duration of the behavioral test. All tests were two-tailed, and significance was determined at *p* < 0.05. All data were analyzed using Prism (version 9, GraphPad, San Diego, CA) and are presented as mean ± SEM in the figures.

## Results

Embryonic ethanol exposure stimulates early behaviors in pre-weanling rats and a later increase in alcohol consumption.

**Figure 1:**
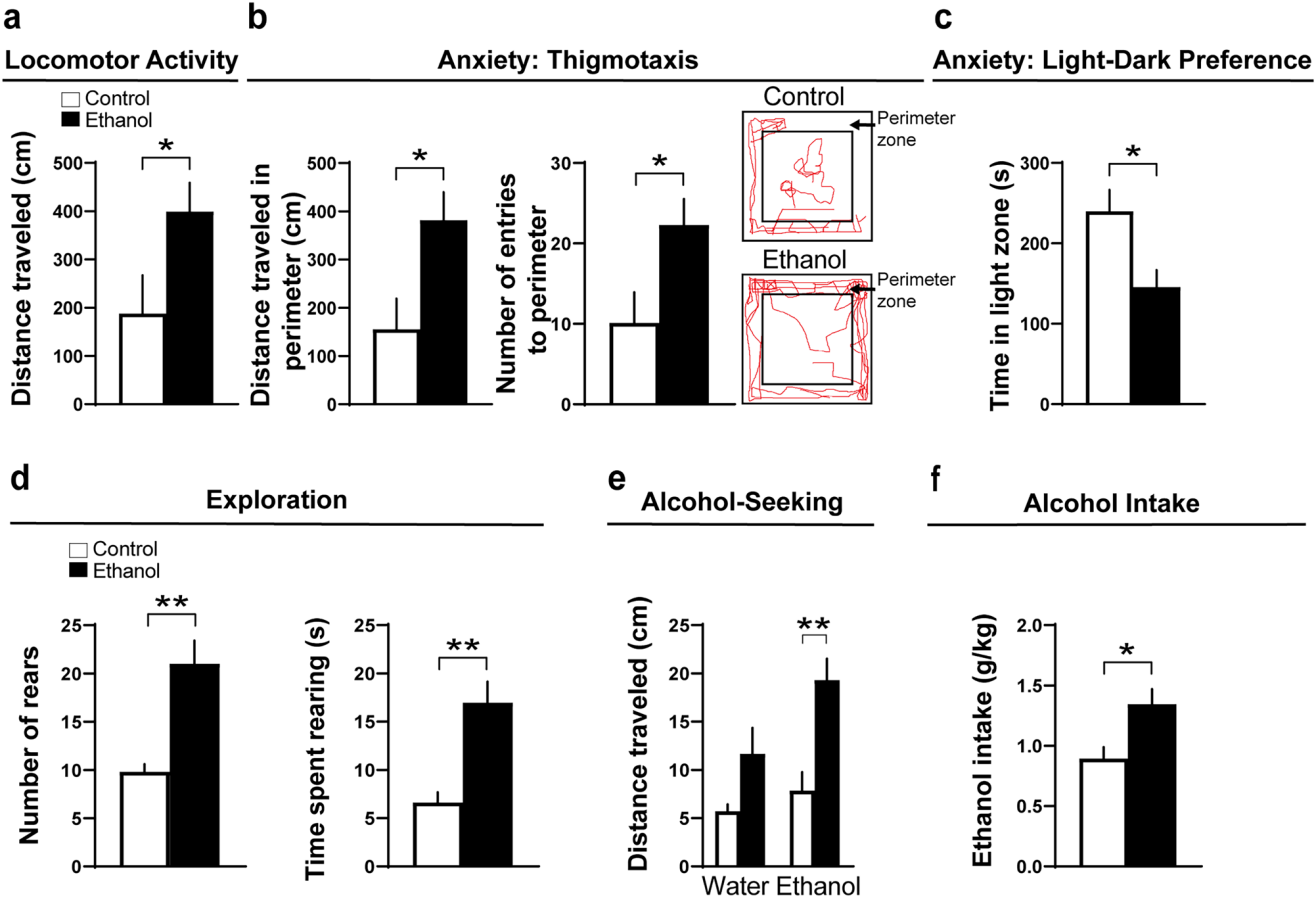
Effects of embryonic ethanol exposure (2 g/kg/day, from E10–E15) compared to control on alcohol-related behaviors, including locomotor activity, anxiety, exploration and alcohol-seeking in pre-weanling female rats and voluntary consumption of alcohol in adolescent female rats. (**a**) Bar graph shows that embryonic ethanol exposure compared to control increases locomotor activity in an activity chamber in 15-day-old rats, as indicated by increased distance traveled (cm) during a 10-min test. (**b**) Bar graphs show that ethanol exposure compared to control increases thigmotaxis in 15-day-old rats, an anxiety-like behavior measured by increased distance traveled in the perimeter (cm) and number of entries into the perimeter within an activity chamber during a 10-min test. Representative activity traces shown in red illustrate the activity of a control and ethanol-exposed rat during the locomotor and thigmotaxis tests. (**c**) Bar graph shows that embryonic ethanol exposure compared to control increases anxiety in 15-day-old rats, as indicated by a decrease in time spent in the light zone during a 10-min light–dark preference test. (**d**) Bar graphs show that embryonic ethanol exposure increases exploratory behavior in 15-day-old rats, as indicated by increased number of rears and time spent rearing in an activity chamber during a 10-min test. (**e**) Bar graph shows that embryonic ethanol exposure compared to control increases alcohol-seeking behavior in 12-day-old rats, as indicated by an increased distance traveled (cm) during a 2-min test down a runway while a cotton ball moistened with 6% ethanol was applied directly in front of the rat’s snout but not while a control cotton ball moistened with water was applied. (**f**) Bar graph shows that embryonic ethanol exposure compared to control in 35-day-old rats increases alcohol consumption as measured using a 20% intermittent-access paradigm. Results are shown as means ± standard errors. *p < 0.05, **p < 0.01.

We first tested in pre-weanling rats the effect of embryonic exposure to ethanol at a low-moderate concentration (2 g/kg/day, from E10 to E15) on early behaviors that are associated with increased alcohol consumption later in life. In the first group of 15-day-old rats, we found that ethanol compared to control increased locomotor activity as measured by distance traveled (t(17) = 2.16, *p* = 0.046) (Fig. 1a) and increased anxiety-like behaviors as measured by a greater distance traveled in the perimeter of the activity chamber (t(16) = 2.63, *p* = 0.019) and number of entries into the perimeter (t(16) = 2.45, *p* = 0.025), as illustrated in the representative activity traces (Fig. 1b). In a second group of rats, ethanol compared to control in a light–dark preference test decreased the amount of time spent in the light zone (t(10) = 2.58, *p* = 0.027), a result also indicative of increased anxiety (Fig. 1c). Exploration was also tested in the first group of 15-day-old rats and was examined by measuring rearing behavior, and the results showed that ethanol increased both the number of rears (t(8) = 4.41, *p* = 0.002) and the time spent rearing (t(8) = 4.24, *p* = 0.002), indicating an increase in exploratory behavior (Fig. 1d). Alcohol-seeking behavior was tested in a third group of rats at 12 days of age, by measuring the distance traveled down the runway after presentation of a cotton ball moistened with either water or 6% ethanol, and this test showed that the ethanol-exposed compared to control rats presented the ethanol-moistened cotton traveled a significantly greater distance toward the cotton ball (t(17) = 3.9, *p* = 0.001), while the ethanol-exposed and control rats presented the water-moistened cotton showed no difference in their distance traveled (t(17) = 2.04, *p* = 0.057) (Fig. 1e). For the final experiment, we used a two-bottle choice test to examine a group of adolescent rats at 35 days of age for their voluntary intake of alcohol and found that the ethanol-exposed rats consumed more alcohol than the control rats (t(6) = 2.87, *p* = 0.028) (Fig. 1f). These results demonstrate that embryonic ethanol exposure at a low-moderate concentration causes a variety of behavioral disturbances in the rat that are evident early in development and are associated with a later increase in alcohol intake during adolescence.

Embryonic ethanol exposure stimulates early behaviors in larval zebrafish and a later increase in alcohol consumption.

**Figure 2:**
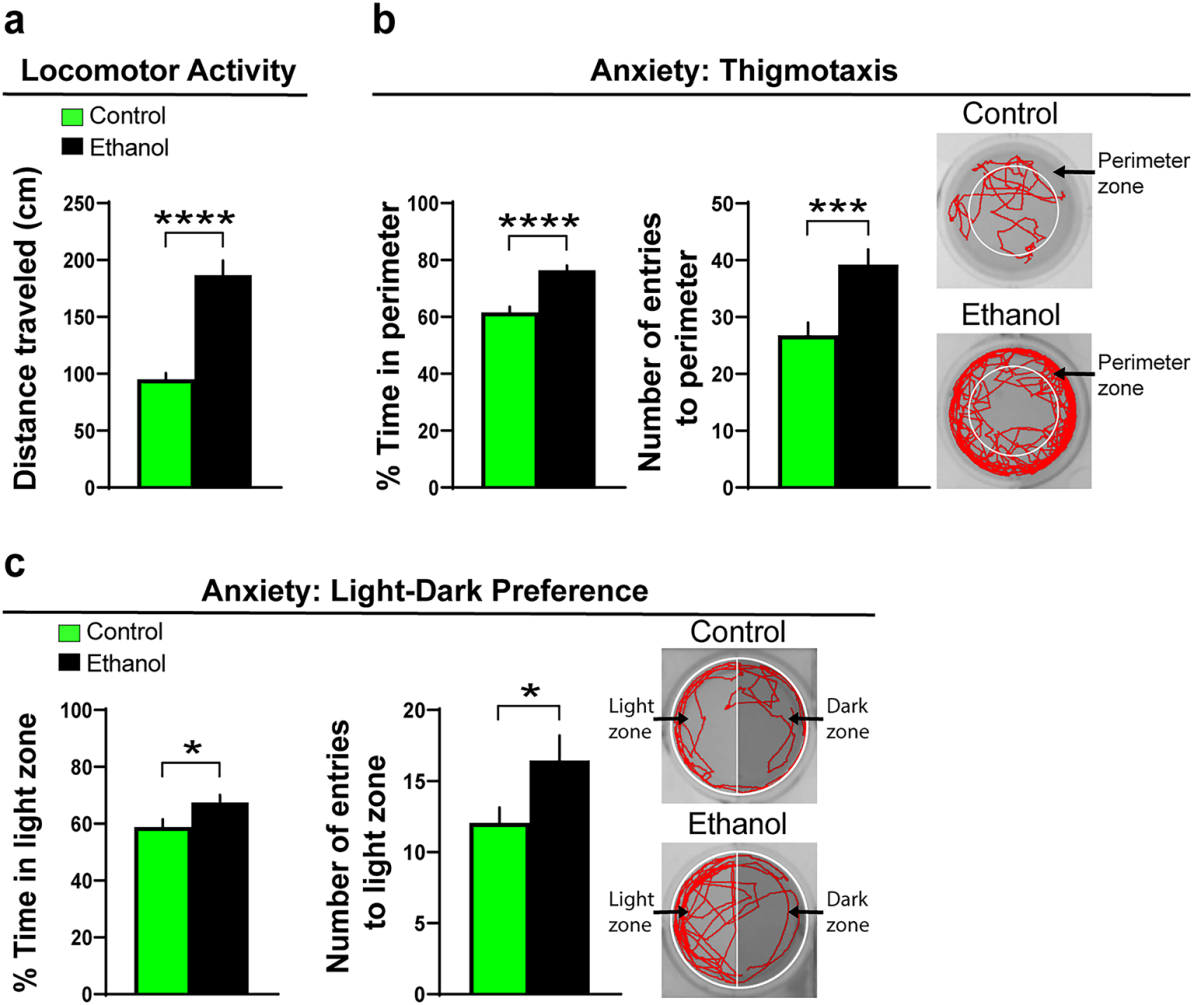
Effects of embryonic ethanol exposure (0.5% v/v, from 22–24 hpf) compared to control on alcohol-related behaviors, including locomotor activity and anxiety-like behaviors, in larval zebrafish (6 dpf). (**a**) Bar graph shows that embryonic ethanol exposure compared to control increases locomotor activity in an open-field environment of a 12-well plate, as indicated by increased distance traveled (cm) during a 20-min test. (**b**) Bar graphs show that ethanol compared to control increases thigmotaxis, an anxiety-like behavior, shown by an increased percent time spent in the perimeter (cm) and number of entries into the perimeter within an open-field environment of a 12-well plate during a 20-min test. Representative activity traces shown in red illustrate the activity of a control and ethanol-exposed zebrafish during the locomotor and thigmotaxis tests, with the white circular outlines illustrating the perimeter zone. (**c**) Bar graphs show that embryonic ethanol exposure compared to control increases anxiety-like behavior, as indicated by an increased percent time spent in and increased number of entries into the light zone during a 20-min light–dark preference test. Representative activity traces shown in red illustrate the activity of a control and ethanol-exposed zebrafish during the light–dark preference test, with the light zone shown on the left half and dark zone shown on the right half of the well. Under visible light conditions, the dark zone is black in color. Results are shown as means ± standard errors. *p < 0.05, ***p < 0.001, ***p < 0.0001. hpf, hours post fertilization; dpf, days post fertilization.

We next sought to determine in the AB strain of zebrafish (6 dpf) if embryonic ethanol exposure in the water at a low-moderate concentration (0.5% v/v, from 22 to 24 hpf) similarly stimulates alcohol-related behaviors at an early age in larvae and increases later alcohol consumption in juvenile zebrafish (30 dpf). Consistent with prior work^24^, we found that ethanol exposure compared to control increased locomotor activity as measured by distance traveled (t(155) = 6.24, *p* < 0.0001) (Fig. 2a) and also increased anxiety-like behaviors as measured by a greater percent time spent in the perimeter (t(155) = 5.99, *p* < 0.0001) and more entries into the perimeter (t(155) = 3.53, *p* = 0.0006), as illustrated by representative swim activity traces (Fig. 2b). Anxiety-like behavior as measured by absolute turn angle was also significantly increased in ethanol-exposed compared to control zebrafish (t(135) = 3.59, *p* = 0.0005). Similarly, in a separate group in the light–dark preference test, ethanol increased the percent time spent in the light zone (t(111) = 2.37, *p* = 0.019) and the number of entries into the light zone (t(111) = 2.125, *p* = 0.036), also indicative of increased anxiety and illustrated by representative swim traces (Fig. 2c).

**Figure 3:**
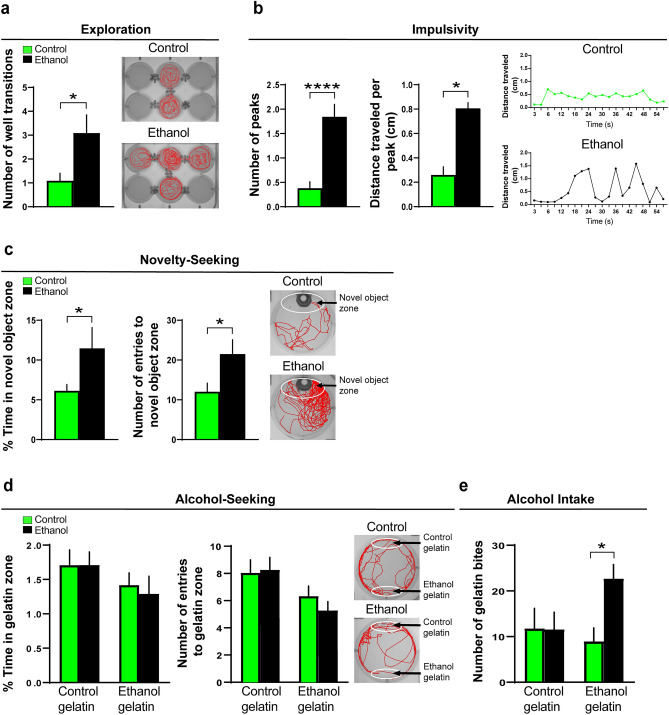
Effects of embryonic ethanol exposure (0.5% v/v, from 22 to 24 hpf) compared to control on alcohol-related behaviors, including exploration, impulsivity, novelty-seeking and alcohol-seeking in 6 dpf zebrafish and voluntary intake of ethanol-gelatin in juvenile zebrafish (30 dpf). (**a**) Bar graph shows that embryonic ethanol exposure compared to control increases exploration in a 6-well environment of a 12-well plate, with channels between the 6 wells allowing for free movement between the wells, as indicated by an increase in number of well transitions during a 20-min test in larval zebrafish. (**b**) Bar graphs show that ethanol compared to control increases motor impulsivity over a 1-min period, shown by an increased number of swimming “peaks” (defined as events when the fish traveled more than 0.5 cm in less than 12 s) and increased distance traveled (cm) during each of these peaks in larval zebrafish. Representative line graphs illustrate in ethanol-exposed (black line) compared to control (green line) zebrafish an increased number of peaks and distance traveled per peak. (**c**) Bar graphs show that embryonic ethanol exposure compared to control increases novelty-seeking behavior in larval zebrafish, as indicated by increased percent time spent in and increased number of entries into the novel object zone during a 20-min test. Representative activity traces shown in red illustrate the activity of a control and ethanol-exposed zebrafish, with the novel-object zone outlined by white at the top of the well surrounding the novel-object consisting of a piece of green-color plastic pipette tip. (**d**) Bar graphs show that embryonic ethanol exposure compared to control has no effect on alcohol-seeking behavior in larval zebrafish, as shown by no change in percent time spent in and number of entries into the ethanol-gelatin as well as control-gelatin zones during the 20-min test. Representative activity traces shown in red illustrate the activity of a control and ethanol-exposed zebrafish, with the control-gelatin zone outlined by white and shown at the top of the well of a 6-well plate and the ethanol-gelatin zone outlined by white and shown at the bottom of the well. (**e**) Bar graphs show that embryonic ethanol exposure compared to control increases alcohol consumption in juvenile zebrafish, as shown by increased number of bites taken of the ethanol-gelatin but not of the control-gelatin. Results are shown as means ± standard errors. *p < 0.05, ***p < 0.0001. *hpf* hours post fertilization, *dpf* days post fertilization.

We next measured the following behaviors, exploration, impulsivity, novelty-seeking and alcohol-seeking in separate groups, that have not previously been well tested in larval zebrafish after embryonic ethanol exposure. Ethanol increased exploratory behavior (t(20) = 2.44, *p* = 0.024), as measured by an increased number of transitions into different wells and shown by representative swim traces (Fig. 3a). It also increased motor impulsivity, as measured by a greater number of swimming peaks (t(59) = 4.93, *p* < 0.0001) and greater distance traveled during each peak (t(59) = 6.63, *p* < 0.0001) and illustrated by representative line graphs (Fig. 3b). Novelty-seeking behavior was evaluated by measuring the percent time spent in the novel object zone and number of entries into the novel object zone, and ethanol exposure was found to increase both the percent time (t(92) = 1.98, *p* = 0.049) and number of entries (t(92) = 2.03, *p* = 0.045) into the novel object zone, as illustrated by representative swim traces (Fig. 3c). Alcohol-seeking behavior was tested by measuring the percent time spent in a zone containing the ethanol-gelatin compared to control-gelatin and the number of entries into these zones, and no differences were detected between the ethanol and control zebrafish for the measures of percent time (t(67) = 0.41, *p* = 0.68) and entries (t(67) = 1.035, *p* = 0.30) into the ethanol-gelatin zone, as well as the measures of percent time (t(67) = 0.002, *p* = 0.99) or entries (t(67) = 0.166, *p* = 0.87) into the control-gelatin zone (Fig. 3d). Lastly, we tested in juvenile zebrafish their voluntary intake of alcohol by measuring the number of bites taken of the ethanol-gelatin versus the control-gelatin and found that the ethanol-exposed compared to control zebrafish consumed significantly more of the ethanol-gelatin (t(14) = 3.15, *p* = 0.14) but a similar amount of the control-gelatin (t(13) = 0.03, *p* = 0.97) (Fig. 3e). Overall, these results show in larval zebrafish, similar to the pre-weanling rats, that embryonic ethanol exposure stimulates a range of behaviors early in development that are positively associated with an increase in consumption of alcohol later in life.

### Optogenetic activation of Hcrt neurons stimulates cfos and early behaviors in larval zebrafish and a later increase in alcohol consumption

**Figure 4:**
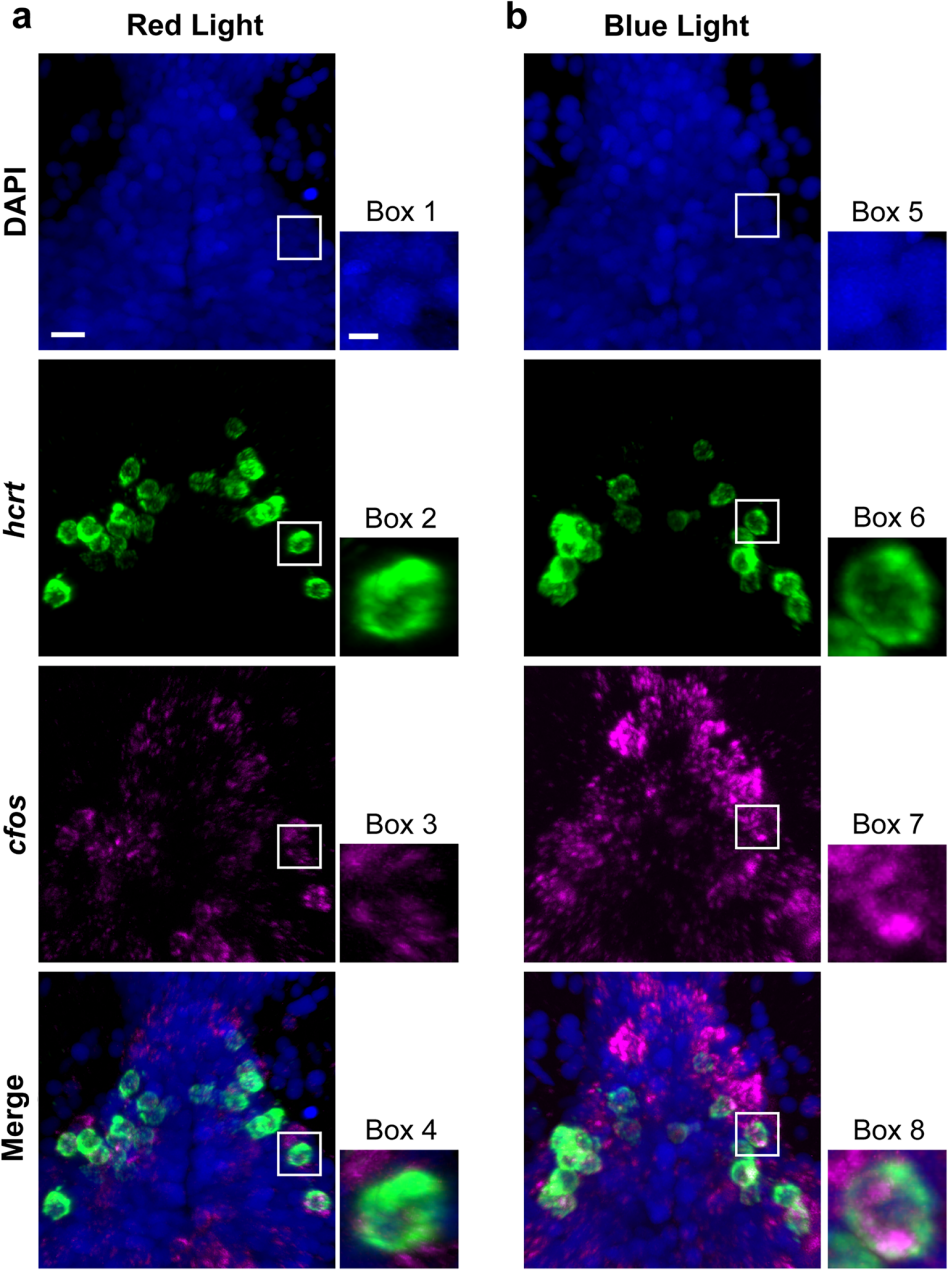
Representative photomicrographs illustrating increased colocalization of transcripts of the immediate early gene cfos, a marker of neuronal activation, within *hcrt* neurons after optogenetic activation with blue light exposure in the brains of transgenic *Hcrt:ChR2-EYFP*^54^ larval zebrafish (6 dpf) using RNAscope staining. (**a**) Representative confocal photomicrograph (×25) illustrates the 6 dpf zebrafish brain in a dorsal/ventral view after 20-min of red-light exposure, a wavelength of light that fails to optogenetically activate Hcrt neurons. Brains were counterstained by DAPI (blue) and labeled for *hcrt* (green) and *cfos* (magenta), with merged photos showing an overlay of each channel and boxes showing single-cell enlargements of DAPI (Box 1), *hcrt* (Box 2), *cfos* (Box 3), and the merge of each channel (Box 4) showing a weak colocalization between *cfos* and *hcrt*. (**b**) Representative confocal photomicrograph (×25) illustrates the 6 dpf zebrafish brain in a dorsal/ventral view after 30-min of blue-light exposure, a wavelength of light that optogenetically activates Hcrt neurons. Brains were counterstained by DAPI (blue) and labeled for *hcrt* (green) and *cfos* (magenta), with merged photos showing an overlay of each channel and boxes showing single-cell enlargements of DAPI (Box 5), *hcrt* (Box 6), *cfos* (Box 7), and the merge of each channel (Box 8) showing strong colocalization between *cfos* and *hcrt*. Scale bars: low-magnification, 10 µm, high-magnification, 2 µm. dpf, days post fertilization.

With embryonic exposure to ethanol shown to stimulate Hcrt neurons in the hypothalamus along with the behavioral changes^24,37^, we next wanted to determine if optogenetic activation of Hcrt neurons in *Hcrt:ChR2-EYFP*^54^ transgenic zebrafish produces effects similar to embryonic ethanol exposure on early behaviors and subsequent alcohol consumption. Our first objective was to confirm in larval zebrafish (6 dpf), by performing RNAscope staining for cfos and examining its colocalization with Hcrt neurons, that exposure to the blue light as described in the “Materials and methods” does in fact activate Hcrt neurons more than the red light. We found that the blue light exposure produced a notable increase in cfos transcripts within the Hcrt neurons relative to the control red light exposure, with approximately 75% of Hcrt expressing cfos in zebrafish exposed to blue light and 25% in zebrafish exposed to red light. This is illustrated in representative photomicrographs and single cell enlargements of the Hcrt neurons in the hypothalamus of the zebrafish exposed to the red light (Fig. 4a) or the blue light (Fig. 4b). This result confirms that blue light administration successfully activates Hcrt neurons in contrast to the red light.

**Figure 5:**
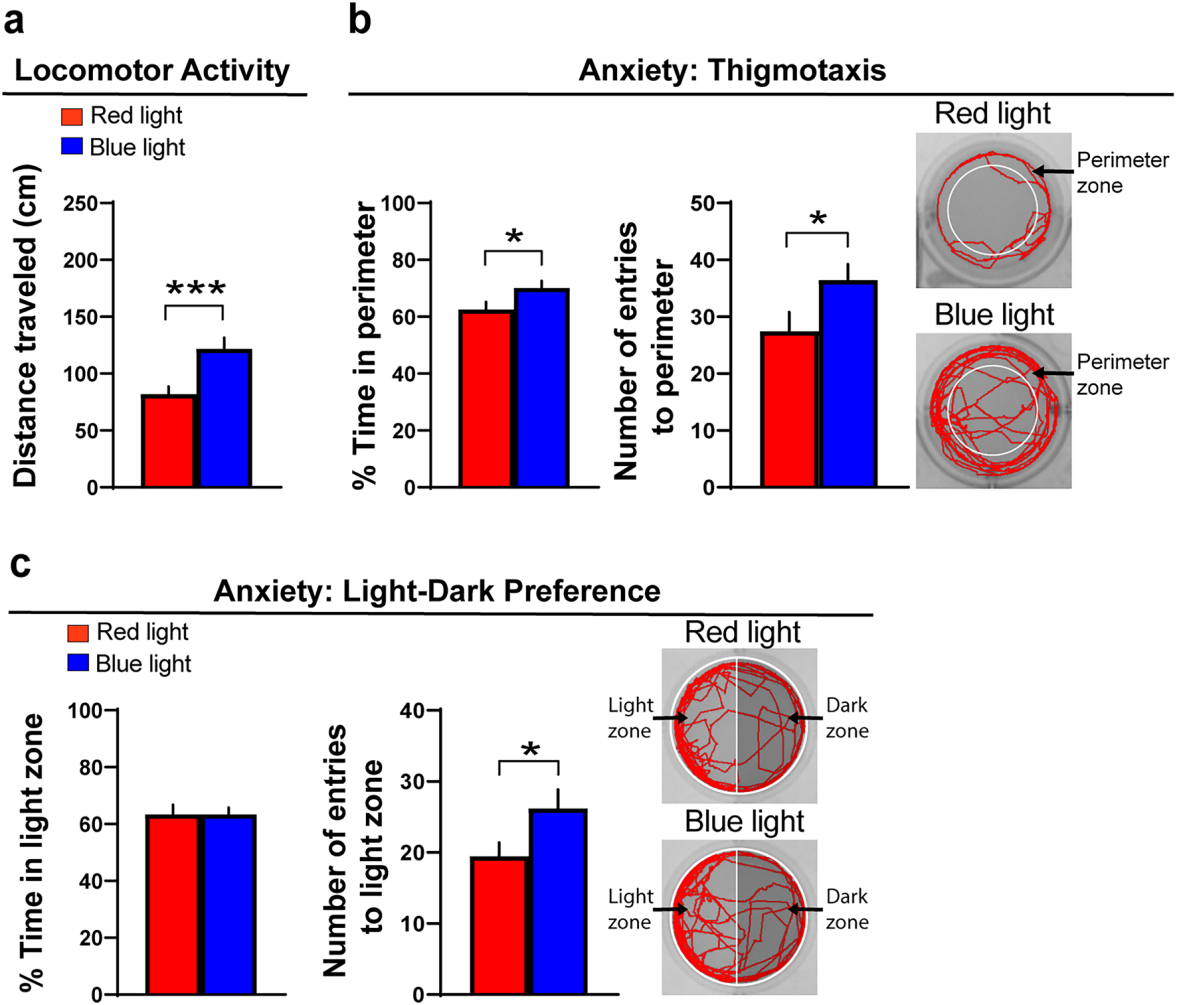
Effects of optogenetic activation of Hcrt neurons on alcohol-related behaviors, including locomotor activity and anxiety, in transgenic *Hcrt:ChR2-EYFP*^54^ larval zebrafish (6 dpf). (**a**) Bar graph shows that optogenetic activation of Hcrt neurons by blue-light compared to red-light exposure increases locomotor activity in an open-field environment of a 12-well plate, as indicated by increased distance traveled (cm) during a 20-min test. (**b**) Bar graphs show that blue-light compared to red-light exposure increases thigmotaxis, an anxiety-like behavior, shown by increased percent time spent in the perimeter (cm) and increased number of entries into the perimeter of an open-field environment of a 12-well plate during a 20-min test. Representative activity traces shown in red illustrate the activity of a red- and blue-light-exposed zebrafish during the locomotor and thigmotaxis tests, with the white circular outlines illustrating the perimeter zone. (**c**) Bar graphs show that blue-light compared to red-light exposure, while having no effect on percent time spent in the light zone, increases the number of entries to the light zone during a 20-min light–dark preference test. Representative activity traces shown in red illustrate the activity of a zebrafish exposed to a red or blue light during the light–dark preference test, with the light zone shown on the left half and the dark zone shown on the right half of the well. Under visible light conditions, the dark zone is black in color. Results are shown as means ± standard errors. *p < 0.05, ***p < 0.001. *hpf* hours post fertilization, *dpf* days post fertilization.

**Figure 6:**
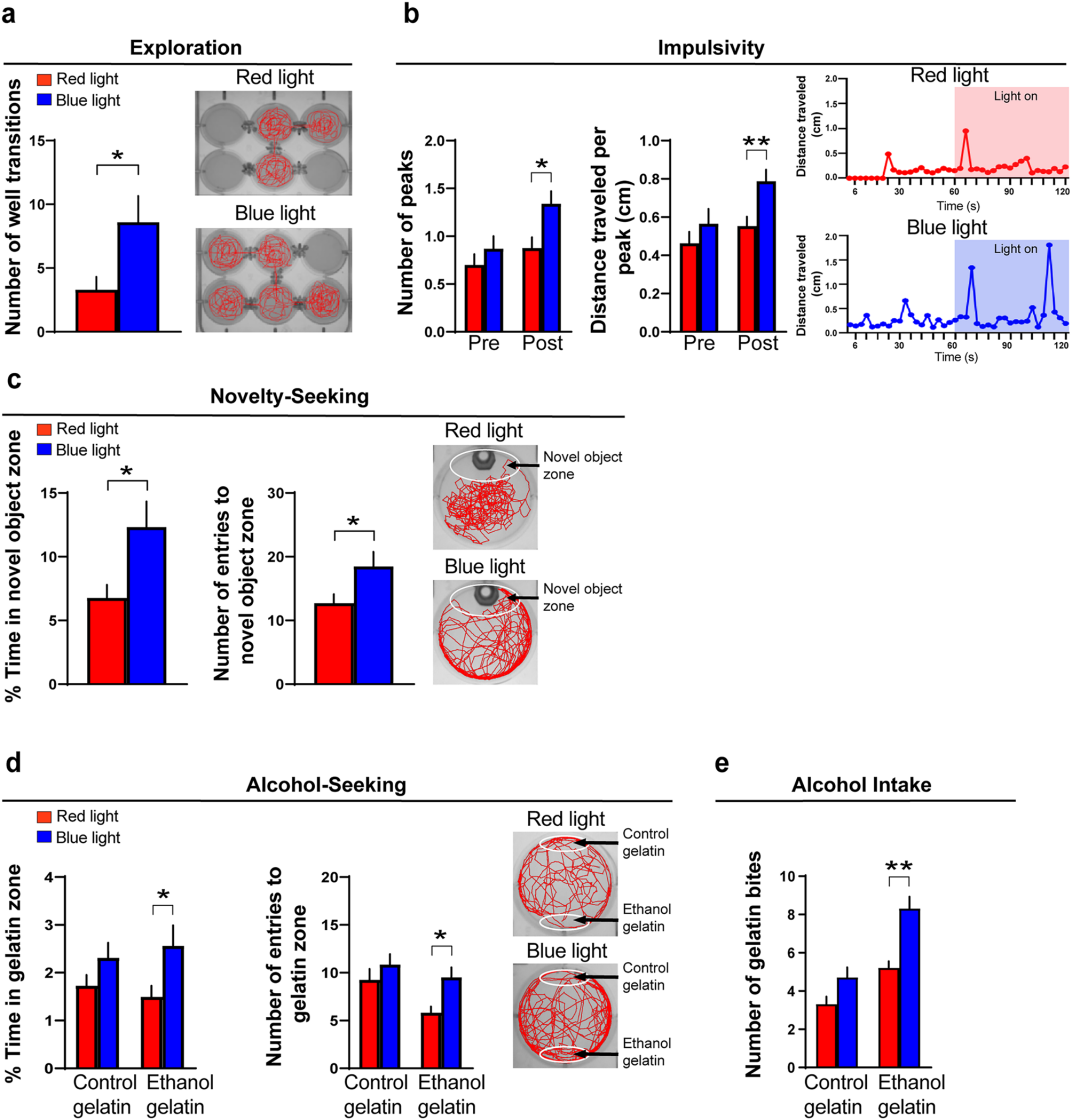
Effects of optogenetic activation of Hcrt neurons in transgenic *Hcrt:ChR2-EYFP*^54^ zebrafish on alcohol-related behaviors, including exploration, impulsivity, novelty-seeking and alcohol-seeking in larval zebrafish (6 dpf) and voluntary intake of alcohol-gelatin in juvenile zebrafish (30 dpf). (**a**) Bar graph shows that optogenetic activation of Hcrt neurons by blue-light compared to red-light exposure increases exploration within a 6-well environment of a 12-well plate (containing channels between the 6 wells that allow for free movement between wells), as indicated by increased number of well transitions during a 20-min test in larval zebrafish. (**b**) Bar graphs show that blue-light compared to red-light exposure increases motor impulsivity in larval zebrafish over a 1-min period post-light but not pre-light exposure, shown by an increased number of swimming “peaks” defined as events when the fish traveled more than 0.5 cm in less than 12 s and by increased distance traveled (cm) during each of these peaks. Representative line graphs show with a red line the distance traveled (cm) by a red-light exposed zebrafish, both before and after the light is turned on, and with a blue line the distance traveled (cm) by a blue-light exposed zebrafish, both before and after the light is turned on. (**c**) Bar graphs show that blue-light compared to red-light exposure increases novelty-seeking behavior in larval zebrafish, as shown by an increase in percent time spent in the novel-object zone and increased number of entries into the novel object zone during a 20-min test. Representative activity traces shown in red illustrate the activity of a red-light and blue-light-exposed zebrafish, with the novel-object zone outlined by white at the top of the well surrounding the novel-object consisting of a piece of green-color plastic pipette tip. (**d**) Bar graphs show that blue-light compared to red-light exposure increases alcohol-seeking behavior in larval zebrafish, as indicated by an increase in percent time spent in and increase in number of entries into the ethanol-gelatin zone but no change in percent time spent in or entries into the control-gelatin zone during the 20-min test. Representative activity traces shown in red illustrate the activity of a red or blue light exposed zebrafish, with the control-gelatin zone outlined by white and shown at the top of the well of a 6-well plate and the ethanol-gelatin zone outlined by white and shown at the bottom of the well. (**e**) Bar graphs show that blue-light compared to red-light exposure increases voluntary alcohol consumption in juvenile zebrafish, as shown by an increased number of bites taken of the ethanol-gelatin but no change in number of bites taken of the control-gelatin. Results are shown as means ± standard errors. *p < 0.05, **p < 0.01. *dpf* days post fertilization.

Measurements of the different behaviors in larval zebrafish revealed effects of optogenetic activation of the Hcrt neurons with the blue light compared to red light (Figs. 5 and 6) that were very similar to those induced by embryonic ethanol exposure compared to control (Figs. 2 and 3). Exposure to the blue light versus red light increased locomotor activity, as measured by distance traveled (t(139) = 3.37, *p* = 0.001) (Fig. 5a), and it also increased anxiety-like behavior, as shown by a greater percent time spent in the perimeter (t(139) = 2.04, *p* = 0.043) and increased entries into the perimeter (t(139) = 2.07, *p* = 0.041) and illustrated by representative swim paths (Fig. 5b). Exposure to blue light versus red light also increased absolute turn angle (t(46) = 2.69, *p* = 0.009), another measure indicative of increased anxiety-like behavior. In the light–dark preference test, the blue light compared to red light, while having no effect on percent time in the light zone (t(97) = 0.009, *p* = 0.99), significantly increased the number of entries into the light zone (t(97) = 2.08, *p* = 0.039), also indicating an increase in anxiety (Fig. 5c).

Blue light compared to red light exposure of the Hcrt neurons similarly stimulated exploratory behavior, as measured by an increased number of transitions into different wells (t(18) = 2.3, *p* = 0.033) and illustrated by representative swim paths (Fig. 6a). It also stimulated motor impulsivity, with no difference between the blue and red light *before* they were turned on observed in the number of swim peaks (t(122) = 0.98, *p* = 0.33) and distance traveled during peaks (t(122) = 1.00, *p* = 0.31) but a significant increase in the blue light compared to red light exposure *after* the lights were turned on observed in both the number of swim peaks (t(122) = 2.67, *p* = 0.017) and distance traveled during the peaks (t(122) = 2.67, *p* = 0.01), as illustrated by representative line graphs (Fig. 6b). Novelty-seeking behavior was also found to be stimulated by optogenetic activation of Hcrt neurons, with blue light compared to red light exposure increasing the percent time in the novel object zone (t(65) = 2.38, *p* = 0.02) and number of entries into the novel object zone (t(65) = 2.08, *p* = 0.042) as illustrated by representative swim paths (Fig. 6c). Alcohol-seeking behavior in larvae was also stimulated by Hcrt activation, with the blue light compared to red light increasing both the percent time in (t(73) = 2.30, *p* = 0.047) and number of entries into (t(73) = 2.80, *p* = 0.012) the ethanol-gelatin zone while having no effect on the percent time in (t(73) = 1.53, *p* = 0.129) or number of entries into (t(73) = 1.00, *p* = 0.319) the control-gelatin zone (Fig. 6d). Lastly, we tested voluntary intake of alcohol in juvenile zebrafish by measuring the number of bites taken of either the control-gelatin or ethanol-gelatin and found that zebrafish exposed to the blue light compared to the red light took more bites of the ethanol-gelatin (t(8) = 4.3, *p* = 0.003) while showing no difference in their number of the bites of the control-gelatin (t(8) = 2.09, *p* = 0.07) (Fig. 6c). Overall, these results show that optogenetic stimulation of Hcrt neurons increases a range of behaviors early in life that is comparable to the behavioral disturbances produced by embryonic ethanol exposure.

## Discussion

Identification of behaviors early in development that can predict susceptibility to developing a future alcohol use disorder is critical for identifying individuals that are most vulnerable. In humans at different ages including childhood, a repertoire of behaviors such as hyperactivity and increased anxiety, novelty-seeking, exploratory behavior, impulsivity, and alcohol-seeking, are shown to be predictive of excess alcohol consumption later in life and are also increased in the offspring of mothers that drink alcohol during pregnancy (see “Introduction”). The present study provides direct evidence that these same behaviors are stimulated by embryonic ethanol exposure at a young age in zebrafish as in rats and are similarly induced in zebrafish by optogenetic activation of Hcrt neurons, a peptide system that is known to stimulate these behaviors and thus may contribute to later life vulnerability to overconsumption of alcohol.

Studies in adolescent and adult rodents have positively linked this repertoire of behaviors to a greater propensity to consume excess alcohol^70–72^ and have also shown these behaviors along with excess alcohol intake to be stimulated by prenatal exposure to ethanol^16–19,53^. Our results here demonstrate these behaviors for the first time in pre-weanling rats prenatally exposed to ethanol at a relatively low concentration. These include a significant increase at 15 days of age of locomotor activity as reported in older ethanol-exposed prepubertal rodents^73^, of anxiety-like behaviors with measures of thigmotaxis and light avoidance as shown with other measures of anxiety in pre-pubertal mice^74^, and of exploratory behavior measured by rearing as shown in juvenile mice^75^. These different behaviors in pre-weanling rats are accompanied by an increase in alcohol-seeking behavior detected at 12 days of age, and they are also followed by an increase in alcohol consumption during adolescence, confirming prior studies in adolescent rats^53^ and consistent with evidence for increased alcohol preference, intake and taste reactivity in ethanol-exposed pre-weanling rats^76,77^. While not measured here, there is one study of impulsivity in pre-pubertal rats suggesting that this behavior is also increased by prenatal ethanol exposure^73^. Together, this evidence demonstrates that behaviors described in older rats embryonically exposed to ethanol are similarly detected at an early age in pre-weanling rats and are associated with a later increase in alcohol consumption during adolescence.

Our results obtained here in larval zebrafish demonstrate a variety of behaviors induced by embryonic exposure to ethanol in the water at a low-moderate concentration, which are similar to those observed in pre-weanling rats and also described in young children (see Introduction). Here we confirm our prior studies showing in larval zebrafish that ethanol exposure increases locomotor activity as measured by total distance traveled and also anxiety as measured by percent time spent in and number of entries into the perimeter of the open field and the light-zone of the light–dark preference test, behaviors associated with an increase in voluntary alcohol intake in adults^22,24–27,78^. Absolute turn angle was also found to be increased by ethanol exposure, a behavior shown to increase in response to fear or anxiety-inducing stimuli^79^. By examining additional behaviors not previously tested in zebrafish after ethanol exposure, our results demonstrate further changes including an ethanol-induced increase in novelty-seeking, as measured by percent time spent in and number of entries into the zone where the novel object is located, and an increase in exploratory behavior, as measured by the number of transitions between wells of six different environments. These behaviors in larval zebrafish are consistent with those induced by acute ethanol exposure in adult zebrafish, including an increase in novelty-seeking^80^ and alterations in exploratory behavior^81^. While impulsive behavior has been investigated primarily in adult zebrafish using the 3 and 5 choice serial reaction time tests requiring associative learning difficult to achieve in larvae^82^, we used here a motor impulsivity test that has been successfully modeled in larval zebrafish^83^ and found this behavior to be increased by embryonic ethanol exposure. Whereas the alcohol-seeking behavior was not affected by ethanol suggesting the ethanol-gelatin may not be rewarding enough for the younger larval zebrafish, an ethanol-induced increase in voluntary consumption of the ethanol-gelatin was observed in the older juvenile zebrafish, consistent with our prior reports in adult zebrafish^22,37^. While clinical evidence and rodent studies have indicated that disturbances in these early behaviors are predictive of alcohol use disorder, longitudinal studies within the same zebrafish and analyses of possible sex differences as previously shown for both zebrafish and rats after sexual maturity ^37,53,84,85^ are needed to provide definitive evidence that this early battery of behavioral changes at a young age are positively correlated with and possibly causally related to an increase in voluntary alcohol intake at later ages.

In addition to these behaviors, embryonic ethanol exposure in larval zebrafish similar to rats is also shown to increase the proliferation, density and expression of Hcrt neurons in the hypothalamus^43,44,86^, and multiple studies show these Hcrt neurons to be involved in controlling numerous aspects of alcohol-related behaviors^24,36,87^. This leads us to question if these Hcrt neurons themselves also have a functional role in mediating the early ethanol-induced behaviors. Here we provide strong evidence in larval zebrafish suggesting that they do, with direct optogenetic stimulation of the Hcrt neurons found to produce the same behaviors induced by embryonic ethanol exposure. The only behaviors previously studied in zebrafish with optogenetic activation of Hcrt neurons are sleep/wake and locomotion^54,88^, and our results here confirm the Hcrt-induced increase in locomotor activity in larval zebrafish. This is consistent with studies in both rodents and zebrafish showing this behavior to be positively related to Hcrt neurons^23,36^ and also to be increased by activation of Hcrt neurons through chemogenetic as well as optogenetic stimulation and decreased by the ablation of Hcrt neurons in animals that were^24^ or were not^45,54,89^ embryonically exposed to ethanol. We further show that anxiety-related behaviors in larval zebrafish, specifically absolute turn angle, thigmotaxis and light-preference, are stimulated by optogenetic activation of Hcrt neurons. This is a novel finding that agrees with other findings, including our recent report in zebrafish showing that ablation of Hcrt neurons blocks anxiety behaviors induced by embryonic ethanol exposure^24^ and evidence in rodents showing that Hcrt injection decreases time spent in the light side of a light–dark test^90^ and chemogenetic and optogenetic stimulation of Hcrt neurons increases anxiety via social interaction^45,91^.

We also find here the less studied behaviors, novelty-seeking, exploration, and impulsivity, to be increased in larval zebrafish by optogenetic stimulation of Hcrt neurons. While little is known about the role of Hcrt neurons in novelty-seeking behavior, there is evidence in methamphetamine-preferring rats for an increase in this behavior in association with increased Hcrt receptor expression in the prefrontal cortex^92^ and also in zebrafish showing central injection of Hcrt to increase novelty-induced locomotion^21^. A relationship of Hcrt to exploratory behavior is suggested by evidence in rodents, showing Hcrt neurons to be active during exploration^93^ and exploratory behavior to be blocked by antagonism of Hcrt receptors^94^. Also, a role for Hcrt in impulsive behavior is suggested by rodent studies, showing that optogenetic stimulation of Hcrt increases impulsivity in the Go/NoGo test and Hcrt antagonism blocks cocaine-induced impulsivity and impulsive responding in a 5-choice serial reaction time test^47,95,96^. While not induced by embryonic ethanol exposure as described above, alcohol-seeking behavior in larval zebrafish is significantly increased by optogenetic stimulation of Hcrt neurons, indicating that direct activation of Hcrt neurons in contrast to ethanol-induced increase in Hcrt expression has sufficient rewarding effects to induce alcohol-seeking behavior at a young age. This positive relationship between Hcrt and both early alcohol-seeking in larval zebrafish and subsequent alcohol consumption in juvenile zebrafish agrees with other reports in adult zebrafish, showing central Hcrt injection to increase voluntary intake of ethanol-gelatin^21^ and the number of Hcrt neurons to be positively correlated with the number of bites taken of ethanol-gelatin^23^. It is also in agreement with studies in rodents, showing Hcrt neuronal activity to be correlated with the intake and preference for alcohol^97^ and both alcohol-seeking behavior and alcohol intake to be induced by central injection of Hcrt^87,98,99^ and reduced by blockade of Hcrt receptors^36,100^. Together, our findings here demonstrate in larval zebrafish a full repertoire of behaviors, which are induced by optogenetic stimulation of Hcrt neurons and similarly by embryonic ethanol exposure that also stimulates endogenous Hcrt, supporting a role for Hcrt neurons in mediating these behavioral effects of ethanol.

In summary, our results here provide evidence suggesting that specific behaviors described in children, which are predictive of excess alcohol consumption later in life and are induced in the offspring by maternal drinking of alcohol during pregnancy, are conserved across species, observed similarly at young ages in zebrafish as well as rats exposed as embryos to ethanol. The use of larval zebrafish here and in our recent studies has allowed us to gain a more in-depth understanding of the role of a particular neural mechanism, specifically hypothalamic Hcrt neurons, in mediating these early behaviors induced by embryonic ethanol exposure that are related to later excess consumption of alcohol. These findings support the use of larval zebrafish as a strong animal model for investigating additional neuronal systems causally related to early, ethanol-induced behavioral disturbances that are predictive of alcohol use disorder later in life.

## Data Availability

The datasets used and/or analyzed during the current study are available from the corresponding author on reasonable request.
